# Tobacco smoking and dopaminergic function in humans: a meta-analysis of molecular imaging studies

**DOI:** 10.1007/s00213-019-05196-1

**Published:** 2019-03-18

**Authors:** Abhishekh H. Ashok, Yuya Mizuno, Oliver D. Howes

**Affiliations:** 1 Psychiatric Imaging Group, MRC London Institute of Medical Sciences Centre (LMS), Du Cane Road, London, W12 0NN UK; 2 Psychiatric Imaging Group, Faculty of Medicine, Imperial College London, Institute of Clinical Sciences (ICS), Du Cane Road, London, UK; 3 Department of Psychosis Studies, Institute of Psychiatry, Psychology & Neuroscience, King’s College London, 16 De Crespigny Park, London, SE5 8AB UK; 4 Department of Neuropsychiatry, Keio University School of Medicine, Tokyo, Japan

**Keywords:** Tobacco smoking, Dopamine, Molecular imaging studies, Meta-analysis

## Abstract

**Rationale:**

About 1.1 billion people smoke tobacco globally and tobacco-related health care costs 1.8% of GDP in many countries. The majority of people are unable to quit smoking despite pharmacological intervention, highlighting the need to understand the pathophysiology associated with tobacco smoking to aid the development of new therapeutics. The reinforcing effects of tobacco smoking are thought to be mediated by the dopamine system. However, the nature of dopamine dysfunction seen in smokers is unclear.

**Objective:**

To determine the nature and robustness of the evidence for dopaminergic alterations in smokers.

**Methods:**

The entire MEDLINE, EMBASE, and PsycINFO databases were searched for studies from inception date to November 18, 2018. In vivo human molecular imaging studies of dopamine measures (dopamine synthesis or release capacity, transporter levels, receptor levels) in tobacco smokers were selected. Demographic, clinical, and imaging measures were extracted from each study and meta-analyses, and sensitivity analyses were conducted.

**Results:**

Fourteen studies met inclusion criteria comprising a total sample of 219 tobacco smokers and 297 controls. The meta-analysis showed a significant reduction in dopamine transporter availability in the smokers relative to controls with an effect size of − 0.72 ([95% CI, − 1.38 to − 0.05], *p* = 0.03). However, there was no difference in D2/3 receptor availability in smokers relative to controls (*d* = −0.16 ([95% CI, − 0.42 to 0.1], *p* = 0.23). There were insufficient studies for meta-analysis of other measures. However, findings from the published studies indicated blunted dopamine release and lower D1 receptor availability, while findings for dopamine synthesis capacity were inconsistent.

**Conclusion:**

Our data indicate that striatal dopamine transporter availability is lower but D2/3 receptors are unaltered in smokers relative to controls. We discuss the putative mechanisms underlying this and their implications.

**Electronic supplementary material:**

The online version of this article (10.1007/s00213-019-05196-1) contains supplementary material, which is available to authorized users.

## Introduction

According to the World Health Organization, estimates of 1.1 billion people smoke tobacco and 6 million deaths/year are linked to tobacco use. Moreover, second-hand smoke exposure is responsible for additional 600,000 deaths (World Drug Report 2015; https://www.unodc.org). It is estimated that tobacco-related health care costs 1.8% of GDP in many countries (Goodchild et al. 2018). Dopaminergic alterations are hypothesized to underlie addictive behavior (Ashok et al. 2017; Di Chiara and Bassareo 2007; Keiflin and Janak 2015; Nutt et al. 2015; Volkow and Morales 2015; Willuhn et al. 2014). Consistent with this, nicotine in tobacco stimulates nicotinic acetyl cholinergic (nACh) receptors leading to dopamine release (Benowitz 2009). Furthermore, preclinical studies show that the acute rewarding effects of nicotine are linked to two primary mechanisms. First, nicotine directly activates VTA dopaminergic neurons, which release dopamine in the nucleus accumbens (NAc). Second, it stimulates nAChR receptors located on the dopaminergic terminals augmenting dopamine release (Di Chiara and Imperato 1988; McGranahan et al. 2011; Zhang et al. 2009; Zhou et al. 2001). Positron emission tomography (PET) and single photon emission computed tomography (SPECT) enable dopaminergic indices to be measured in vivo in humans (Kim et al. 2013). A number of studies have investigated dopamine release, dopamine transporter, and dopamine receptor levels in smokers. However, the robustness of findings remains unclear and, to our knowledge, there has not been a previous meta-analysis of these findings. Thus, we aimed to synthesize the PET and SPECT imaging findings on dopaminergic function in smokers and to consider their implications for therapeutics. We group findings into studies of dopamine synthesis, dopamine release, dopamine transporter availability, and dopamine receptor availability. We focused on the whole striatum as it is richly innervated with dopaminergic neurons and reliably quantified with PET and SPECT in humans (Ashok et al. 2017; Egerton et al. 2010; Howes et al. 2012).

## Methods

### Study selection

To be included in the meta-analysis, an article needed to investigate the striatal dopaminergic system in human tobacco smokers. The MEDLINE, EMBASE, and PsycINFO databases were searched from inception date to November 18, 2018, for relevant papers without language restrictions. The electronic searches using EMBASE and PsycINFO were carried out together using Ovid. The following keywords were used: “(Positron Emission Tomography OR PET OR Single photon emission tomography OR SPET OR Single Photon Emission Computed Tomography OR SPECT) AND (dopamine OR dopamine release OR dopamine synthesis OR dopamine availability OR dopamine transporter OR dopamine reuptake OR dopamine receptor) AND (smoking OR nicotine OR nicotine dependence OR tobacco dependence)”. In addition, the reference lists in the included studies and relevant review papers were screened to search for additional studies. Further details of study selection are provided in the supplementary Fig. 1.

### Inclusion and exclusion criteria

The inclusion criteria were as follows: 1) original molecular imaging studies that indexed dopamine receptors, or dopamine transporters and/or dopamine release or synthesis; 2) included a group of regular (daily) tobacco smokers; and 3) reported data for the whole striatum or a striatal sub-region. We excluded studies which did not have a healthy control group or that included subjects with CNS co-morbidity. For studies with an overlap in participants, we included the study with the largest sample size without potentially missing any subject and excluded the smaller study from the meta-analysis to avoid duplication of subjects, consistent with previous molecular imaging meta-analyses (Ashok et al. 2017; Kambeitz et al. 2014).

### Data extraction

The primary outcome measure was the difference in the dopaminergic imaging index between smokers and controls. The following variables were extracted from all the studies: authors, year of publication, subject characteristics of the control and smokers (group size, age, sex, substance use characteristics, comorbid substance abuse, method of abstinence confirmation, duration of abstinence, diagnosis), imaging characteristics (method, radiotracer, scanner type and resolution), route of administration of drug challenge, and modeling method.

### Data analysis

The main outcome measure was the effect size calculated as Hedges’ g for the dopaminergic index for the whole striatum in the smokers using a random effects model. Publication bias was assessed by visual inspection of funnel plots and tested with a regression test for funnel plot asymmetry (mixed-effects meta-regression model). Trim and fill analysis was conducted to impute potentially missing studies. Heterogeneity was estimated using the *I*^2^ value (*I*^2^ values < 50% indicate low to moderate heterogeneity, whereas *I*^2^ > 50% indicate moderate to high heterogeneity). A significance level of *p* < 0.05 (two-tailed) was taken as significant.

## Results

The literature search yielded 610 records, from which we identified 19 relevant papers (see Supplementary Fig. 1 for the PRISMA diagram of the literature search). Fourteen of the 19 studies met criteria for inclusion in the quantitative synthesis. There was an insufficient number of studies for the meta-analysis of the D1 receptor (*n* = 2) and dopamine synthesis (*n* = 3) (Table 1).

**Table 1:**
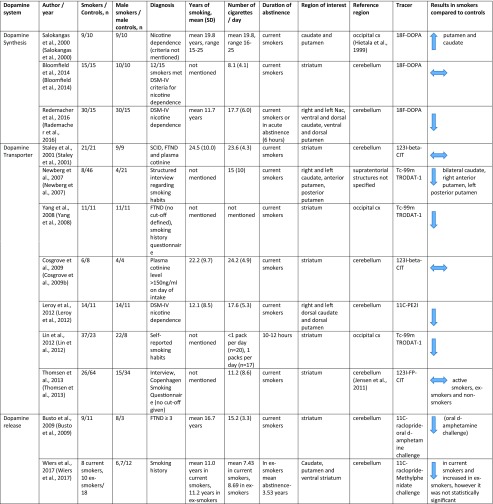

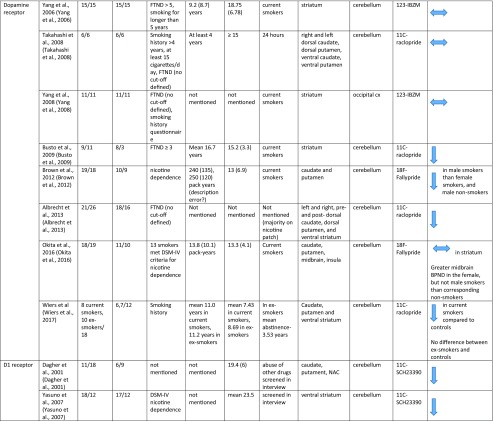
Molecular imaging studies on dopaminergic function in smokers

### Dopamine transporter

**Fig. 1:**
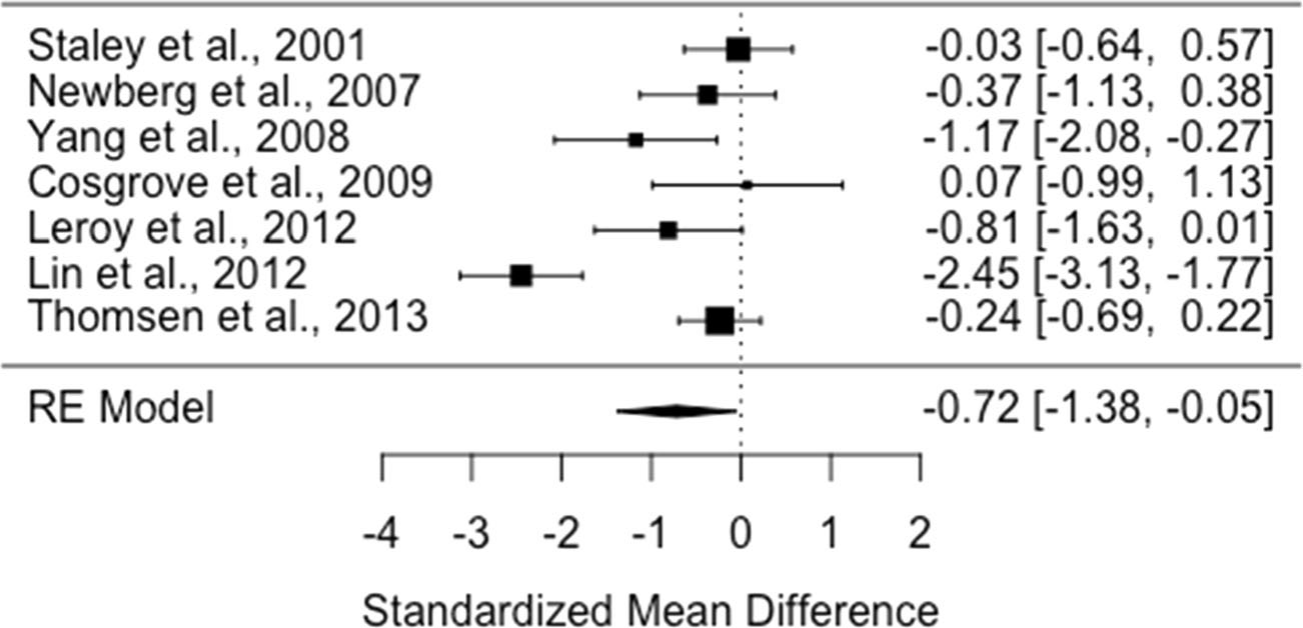
Studies of dopamine transporter availability in tobacco smokers relative to controls. The forest plot shows the effects sizes estimated using a random effects model and 95% confidence intervals of the difference between smokers and controls. There was an overall significant decrease in the dopamine transporter availability in smokers relative to controls with a moderate to large effect size (− 0.72 [95% CI, −1.38 to − 0.05], *p* < 0.05)

There were seven studies assessing dopamine transporter availability in 123 smokers and 184 healthy controls (Cosgrove et al. 2009b; Leroy et al. 2012; Lin et al. 2012; Newberg et al. 2007; Staley et al. 2001; Thomsen et al. 2013; Yang et al. 2008). The meta-analysis showed a significant reduction in dopamine transporter availability in the smoker relative to control groups with an effect size of − 0.72 ([95% CI, − 1.38 to − 0.05], *p* = 0.03) (Figure 1).

### Heterogeneity and sensitivity analyses

The *I*^2^ value was 84% (95% CI, 60–97%), indicating high heterogeneity between studies. The regression test for funnel plot asymmetry was not significant (*t* = −0.5, df = 5, *p* = 0.64). However, visual inspection of the funnel plot revealed asymmetry, indicating possible publication bias. The trim-and-fill analysis indicates two missing studies on the left side of the funnel plot (Supplementary Figure 2). However, the results remained significant after correcting for putatively missing studies (adjusted effect size = −1.0, (95%CI, − 1.6 to −0.37), *p* < 0.01).

### Dopamine receptor availability

**Fig. 2:**
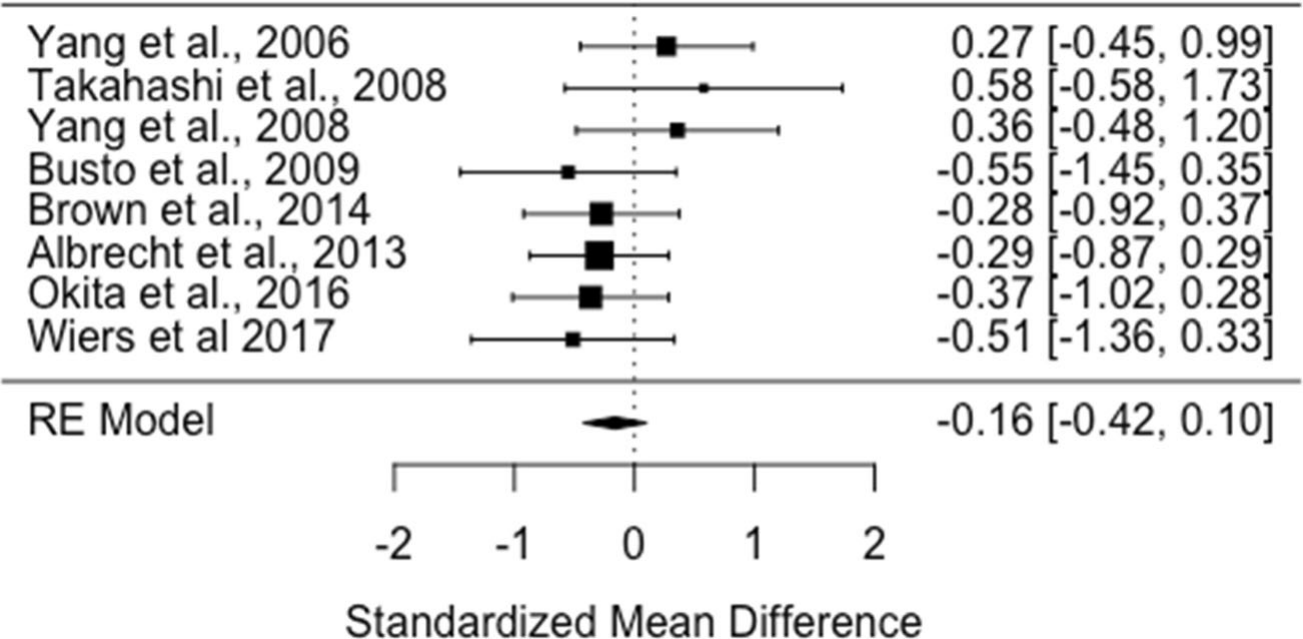
Studies of dopamine D2/3 receptor availability in tobacco smokers relative to controls. The forest plot shows the effect sizes estimated using a random effects model and 95% confidence intervals of D2/3 receptor binding potentials. There was no significant difference in dopamine receptor availability in smokers compared to controls (− 0.16 [95% CI, − 0.42 to − 0.1], *p* > 0.05)

There were eight studies assessing dopamine receptor availability in 107 smokers and 124 healthy controls (Albrecht et al. 2013; Brown et al. 2012; Busto et al. 2009; Okita et al. 2016; Takahashi et al. 2008; Wiers et al. 2017; Yang et al. 2006, 2008). The meta-analysis revealed no significant difference in D2/3 receptor availability in smokers relative to controls with an effect size of − 0.16 ([95% CI, − 0.42 to 0.1], *p* = 0.23) (Figure 2).

### Heterogeneity and sensitivity analyses

The *I*^2^ value was 0% (95% CI, 0–79%), indicating heterogeneity was low. The regression test for funnel plot asymmetry was not significant (*t* = −1.1, df = 6, *p* = 0.32). However, a visual inspection of the funnel plot revealed asymmetry, indicating possible publication bias. The trim-and-fill analysis indicated that there were potentially one missing studies on the left side of the funnel plot (Supplementary Fig. 3). Nevertheless, the summary effect size remained non-significant after correcting for these putatively missing studies (corrected effect size: − 0.2 [95% CI, − 0.45 to 0.05]; *z* = −1.5; *p* = 0.12).

### Dopamine D1 receptor availability

Two studies reported D1 receptor availability, which used [11C] SCH23390 to compare smokers with controls (Dagher et al. 2001; Yasuno et al. 2007). Both studies reported significant reductions in D1 receptor availability in smokers compared to controls.

### Dopamine synthesis

Meta-analysis was not conducted as there were only three studies. There is a substantial discrepancy in the dopamine synthesis capacity in smokers. Salokangas et al. 2000 demonstrated higher dopamine synthesis in heavy smokers relative to controls (Salokangas et al. 2000), Bloomfield et al. 2014 showed no change in moderate smokers (Bloomfield et al. 2014b), while Rademacher et al. 2016 demonstrated lower dopamine synthesis relative to controls in a sample of heavy smokers who met criteria for dependence (Rademacher et al. 2016). Interestingly, reduced dopamine synthesis in heavy smokers normalized after 3 months of abstinence (Rademacher et al. 2016). Overall, the effect of smoking on dopamine synthesis is unclear and further studies in both moderate and heavy smokers are needed.

### Dopamine release

Seminal work in the 1990s developed paradigms to assess dopamine release in vivo using molecular imaging (Breier et al. 1999; Laruelle et al. 1995). This and subsequent work has determined that the reduction in striatal binding of radiotracers such as [11C]-raclopride following the administration of amphetamine or methylphenidate is closely related to the magnitude of dopamine release (Abi-Dargham et al. 2009; Egerton et al. 2009). Only two studies investigated the dopamine release following amphetamine or methylphenidate. One study showed smokers had lower dopamine release compared to healthy control (Busto et al. 2009), while the other study reported a trend level reduction in dopamine release in smokers (Wiers et al. 2017). Thirteen studies measured dopamine release following nicotine administration during or prior to the scan (Barrett et al. 2004; Brody et al. 2010, 2009b, 2006b, 2004; Cosgrove et al. 2014; Domino et al. 2013; Le Foil et al. 2014; Montgomery et al. 2007; Scott et al. 2007a; Takahashi et al. 2008; Weinstein et al. 2016; Wing et al. 2015). The majority of these studies did not have control arm and there was substantial variation in the study design with respect to route of administration, scanning, and nicotine administration duration. Thus, these studies did not meet our inclusion criteria for meta-analysis. Two studies (Barrett et al. 2004; Montgomery et al. 2007) did not report change in binding potential while other studies reported 7–27% reduction in the binding potential (Brody et al. 2006b, 2004; Le Foil et al. 2014; Scott et al. 2007b; Takahashi et al. 2008).

## Discussion

Our main findings are that dopamine transporter availability is reduced with a medium to large effect size and that D2/3 receptor availability in unaltered in smokers compared to healthy controls (Hedges’ g: − 0.72 and − 0.16 respectively). Our sensitivity analyses of the dopamine D2/3 receptor availability showed consistent results, and we noted low heterogeneity. However, there was significant heterogeneity in the dopamine transporter finding.

There were insufficient studies to meta-analyze findings on dopamine synthesis in smokers, and the results of studies were inconsistent, indicating further, large studies are needed to determine if dopamine synthesis capacity is altered by smoking. Similarly, there were too few studies for meta-analyses of dopamine release or D1 receptor levels, although findings indicated blunted dopamine release and lower D1 levels in smokers. However, there were only two studies for each of these dopamine measures, and the studies had small sample sizes. Thus, while results indicate blunted dopamine release and D1 levels in smokers, further studies are needed before the consistency and robustness of these alterations can be determined.

Our finding that D2/3 receptor levels are unaltered is consistent with post-mortem evidence, which also shows unaltered D2 receptor levels in smokers (Court et al. 1998). However, our finding of reduced dopamine transporter availability is not consistent with a human post-mortem study which found DAT levels to be unaltered in smokers (Court et al. 1998). This discrepancy between our in vivo findings and the human post-mortem study could reflect changes in post-mortem or differences in the techniques. This study used a [3H]mazindol binding assay, and evidence indicates mazindol binds to serotonin, norepinephrine, and dopamine transporters (Kung et al. 1995; Owens et al. 1997). Thus, binding to serotonin and norepinephrine transporters could have confounded the post-mortem findings, although it should be appreciated that this is also a potential issue for some PET radiotracers. In contrast, there is some preclinical evidence that DAT function is reduced after nicotine administration as measured by dopamine reuptake in the nucleus (Danielson et al. 2014), and that stimulation of acetylcholine receptor suppresses DAT activity (Huang et al. 1999), consistent with our findings.

### Strengths and limitations

Similar to other meta-analyses of psychiatric imaging studies, there are variations between studies in terms of co-morbid use of other substances such as alcohol and variation in methods, in the radiotracer used, scanners and different definition of the striatum (Ashok et al. 2017; Howes et al. 2012) (Table 1 and Supplementary Table 1). The studies included in the meta-analysis used tracers such as TRODAT and beta-CIT to quantify DAT, but a potential issue is that these tracers also have affinity for serotonin transporters (de Win et al. 2005; Dresel et al. 1999; Stengler-Wenzke et al. 2006). This, coupled with the experimental variables discussed above, could contribute to the relatively high inconsistency we found in the dopamine transporter findings. Nevertheless, the random effects model we used allows for variations in effects.

An association has been reported between a variable number tandem repeats (VNTR) polymorphism of DAT gene (SLC6A3) and DAT availability (Heinz et al. 2000) and this was replicated in an in vitro study (VanNess et al. 2005), although subsequent studies have been inconsistent (Faraone et al. 2014). Variation in other genes related to dopamine function has also been associated with differences dopamine imaging measures (Brody et al. 2006b; Dahoun et al. 2018). As the majority of the studies included in our meta-analysis have not reported genotype data, a potential confounding effect of genetic differences between groups influencing the results cannot be ruled out. Of course, the effects of these genetic variants on the dopamine system may be a mechanistic link that explains why some people are vulnerable to become tobacco smokers. In view of this, it would be useful for future studies to genotype participants for gene variants known to impact on the dopamine system where possible.

A few studies did not explicitly exclude comorbid substance use or report current and past substance use in subjects (see Supplementary Table 1 for details). A meta-analysis has shown that stimulant users have lower dopamine receptor, transporter, and release (Ashok et al. 2017) and alcohol, cannabis, and opiate use may also alter the dopaminergic system (Ashok et al. 2017; Bloomfield et al. 2016; Bloomfield et al. 2014a; Nutt et al. 2015). As such, it is possible that the inclusion of subjects with comorbid stimulant or other substance use could be a confound in some studies. However, the majority of the studies explicitly excluded subjects with current comorbid substance use, suggesting that comorbid substance use is unlikely to have had a major effect on our findings. Nevertheless, it would be useful for all future studies to either exclude comorbid substance use or report it to enable this possibility to be investigated further.

As shown in Table 1, there is substantial variation between studies in the duration of abstinence before the scan. Microdialysis studies have shown that dopamine release reaches peak between 20 and 40 min after nicotine administration and returns to baseline after 60 min (Marshall et al. 1997; Mifsud et al. 1989). As acute smoking-induced dopamine release can displace binding of radiotracers such as [11C] raclopride (Brody et al. 2006b), recent smoking could be a potential confound. In addition, in a study which compared D2 receptor availability and release in a group of current and ex-smokers, prolonged abstinence was shown to normalize the dopaminergic alteration (Wiers et al. 2017). Future longitudinal studies would be useful to determine the temporal course of dopaminergic alterations and abstinence from smoking. A general limitation of the literature is that there are few studies with large sample sizes in dopamine synthesis, release, and D1 receptor availability. Thus we could not meta-analyze these findings.

### Implications for the understanding of the neurobiology of tobacco smoking

Preclinical studies using in vivo micro-dialysis have shown that the acute administration of nicotine increases extra-cellular dopamine concentrations in the striatum, specifically in nucleus accumbens (Damsma et al. 1988; Gaddnas et al. 2001; McCallum et al. 2012; Watkins et al. 2000). Knockout animal models have shown that nicotinic acetylcholine receptor stimulation is required for this effect (Marubio et al. 2003; Picciotto et al. 1999). Human in vivo imaging studies also show that acute exposure to nicotine leads to increased synaptic dopamine, despite substantial variation in study methodology (Barrett et al. 2004; Brody et al. 2010, 2009b, 2004; Cosgrove et al. 2015; Domino et al. 2012, 2013; Le Foll et al. 2014; Montgomery et al. 2007; Scott et al. 2007b). Moreover, molecular imaging studies have shown that occupancy of the nicotinic acetylcholine receptor is associated with the subjective hedonic response of smoking (Brody et al. 2009a, 2011, 2006a, 2014, 2013; Cosgrove et al. 2009a; Cosgrove et al. 2012; Dubroff et al. 2015; Lotfipour et al. 2012; Staley et al. 2006). Thus, there is converging evidence from pre-clinical and human studies that nicotinic acetylcholine receptor-induced dopamine release occurs acutely with tobacco smoking.

In contrast, preclinical studies have shown that chronic (3–4 weeks) nicotine exposure reduces basal dopamine level (Zhang et al. 2012). Electrophysiological studies are consistent with these findings and report that that chronic nicotine administration reduces the firing rate of A10 dopamine neurons (Rasmussen and Czachura 1995). However, D2 receptor availability remains unaltered in chronically nicotine-treated rats (Kirch et al. 1992). Our findings in humans on D2 receptor availability in smokers are consistent with these findings.

Two basic models are possible to account for our findings of reduced dopamine transporter and unaltered D2 receptor availability. The first is that reduced transporter levels may be compensatory in response to reduced tonic dopamine levels or other dopaminergic changes in the synapse. However, normal D2/3 receptor levels are less easy to understand in the context of presynaptic reductions, as it would be expected that they would increase in response to reduced tonic dopamine levels, albeit longitudinal studies are needed to test whether there is a change in D2/3 receptor levels with chronic smoking in humans.

The second model is that lower dopamine transporter levels may underlie the pathoetiology of smoking, and precede its onset. Thus, individuals at risk of smoking may have lower dopamine transporter levels secondary to genetic and environmental risk factors. Lower transporter levels may then mean that the acute effects of smoking, including dopamine release, are larger, potentially making these individuals more vulnerable to become regular users. Future longitudinal human PET studies are needed to investigate changes in the dopamine transporter levels, and other aspects of the dopamine system, prior to and during nicotine addiction, and following cessation to test these models. This will also potentially identify biomarkers to guide treatment and predict outcomes.

## Conclusions

There is evidence for lower dopamine transporter availability with a moderate to large effect size but normal D2 dopamine receptor availability in smokers. These findings identify dopamine transporter abnormalities as either involved in the pathophysiology of tobacco dependence or as a biological response to long-term exposure to tobacco. Further studies are needed to determine the nature of alterations in other aspects of the dopamine system, and whether there are longitudinal changes in dopamine transporter levels during the acquisition of a smoking habit.

## Electronic supplementary material

ESM 1 (DOCX 1227 kb)

Table S1 (DOCX 32.6 kb)
