# Supplementary material for: Tobacco smoking and dopaminergic function in humans: a meta-analysis of molecular imaging studies
Source: Psychopharmacology (Berl). 2019 Mar 18;236(4):1119–29. doi: 10.1007/s00213-019-05196-1 (PMC6591186; doi:10.1007/s00213-019-05196-1)
Supplement: Supplementary file 1 — (DOCX 1227 kb) [file 213_2019_5196_MOESM1_ESM.docx]

**Supplementary Material**

Ashok AH, Mizuno Y, Howes OD. Tobacco smoking and dopaminergic function in humans: a meta-analysis of molecular imaging studies.

**Supplementary Figure 1**. Flowchart showing the inclusion of studies for the systematic review and meta-analysis on dopaminergic function in smokers

**Supplementary Figure 2**. Funnel plot showing two missing studies on the left in studies which assessed dopamine transporter in smokers

**Supplementary Figure 3**. Funnel plot showing one missing study on the left in studies which assessed dopamine receptor in smokers

**Supplementary Table 1**. Subject characteristics of the PET studies

**Supplementary Figure 1.** Flowchart showing the inclusion of studies for the systematic review and meta-analysis on dopaminergic function in smokers

Records identified in electronic search (n=610)

(Embase, MEDLINE, and PsycINFO combined in Ovid)

## **Screening**

## **Included**

## **Eligibility**

## **Identification**

Records after duplicates removed (n=399)

Titles and record types screened (n=399)

Records excluded (n=327)

Full-text assessed for eligibility (n=50)

Records excluded for the following reasons:

No healthy, non-smoker control group (n=19)

Additional review article (n=3)

Simulated study (n=3)

Only healthy, non-smoker participants (n=1)

Co-morbid attention deficit hyperactivity disorder (n=1)

Co-morbid Parkinson’s disease (n=1)

Co-morbid schizophrenia (n=1)

No extractable data (n=2)

Studies included in the qualitative synthesis

(n=19)

Full-text articles remaining (n=19)

Additional publications identified by hand-search

of reference lists (n=0)

Records excluded (n=22)

Abstracts screened (n=72)

Dopamine synthesis (n=3)

Dopamine release (n=2)^*1^

D1 receptor (n=2)

Studies included in the meta-analysis (n=14)

Dopamine D2/3 receptor (n=8) ^*1^

Dopamine transporter (n=7) ^*2^

Dopamine synthesis (n=3)

^*1^ Studies of dopamine release were included in the meta-analysis of dopamine D2/3 receptor, ^*2^ One study by Yang et al., 2008 was included in the meta-analysis of both dopamine D2/3 receptor and dopamine transporter

**Supplementary Figure 2**. Funnel plot showing two missing studies on the left in studies which assessed dopamine transporter in smokers


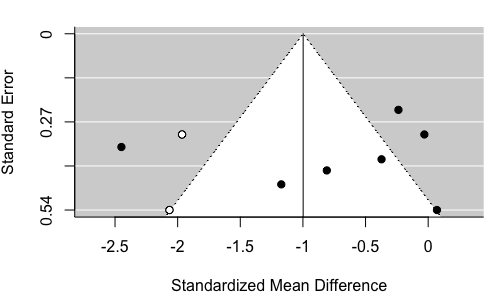


**Supplementary Figure 3**. Funnel plot showing one missing study on the left in studies which assessed dopamine receptor in smokers


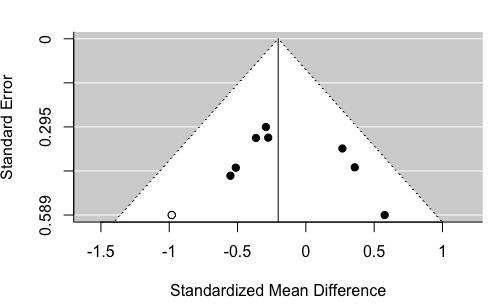


**Supplementary Table 1.** Subject characteristics of the PET studies

| Dopamine system | Author/year | Age, smokers mean (SD) | Age, controls mean (SD) | FTND values | Level of CO concentration pre-scan (yes/no) | Was co-morbid substance abuse excluded? |
| --- | --- | --- | --- | --- | --- | --- |
| Dopamine Synthesis | Salokangas et al., 2000 | 37.1 (3.4) | 36.8 (4.1) | not mentioned | no | not mentioned |
|  | Bloomfield et al., 2014 | 29.9 (10.3) | 29.5 (11.1) | not mentioned | no | Yes- by clinical interview and urine drug screen |
|  | Redemacher et al., 2016 | 28.4 (7.1) | 27.9 (7.5) | 4.9 (1.5) | yes, but data not given | Yes- by clinical interview |
| Dopamine Transporter | Staley et al., 2001 | 40.4 (10.7) | 40.0 (11.0) | 5.1 (1.7) | no | Yes- by clinical interview and urine drug screen |
|  | Newberg et al., 2007 | 41.8 (17.8) | 37.5 (15.7) | not mentioned | no | not mentioned |
|  | Yang et al., 2008 | 28.7 (8.8) | 27.1 (6.2) | 5.6 (1.0) | no | Yes- by clinical interview |
|  | Cosgrove et al., 2009 | 37.8 (12.6) | 38.8 (8.3) | 5.8 (2.5) | no | Yes- by clinical interview and urine drug screen |
|  | Leroy et al., 2012 | 27.0 (8.4) | 30.2 (8.0) | 4.4 (2.2) | no | Yes- by clinical interview and urine drug screen |
|  | Lin et al., 2012 | 35.79 (8.43) | 40.43 (11.22) | not mentioned | no | not mentioned |
|  | Thomsen et al., 2013 | 47.2 (19.4) | 51.7 (18.5) | not mentioned | no | Yes- by clinical interview and urine drug screen |
| Dopamine release | Busto et al., 2009 | 36.8 (13) | 27.5 (4.0) | 4.9 (1.2) | no | Yes- by clinical interview and urine drug screen |
|  | Wiers et al., 2017 | 31.5 (8.0) | 33.9 (9.1) | not mentioned | yes | Yes- by clinical interview and urine drug screen |
| Dopamine receptor | Yang et al., 2006 | 33.3 (10.9) | 33.2 (11.7) | 5.8 (0.92) | yes | Yes- by clinical interview |
|  | Takahashi et al., 2008 | 25.8 (2.6) | 23.7 (2.7) | data not given | no | Yes- by clinical interview |
|  | Yang et al., 2008 | 28.7 (8.8) | 27.1 (6.2) | 5.6 (1.0) | no | Yes- by clinical interview |
|  | Busto et al., 2009 | 36.8 (13) | 27.5 (4.0) | 4.9 (1.2) | no | Yes- by clinical interview and urine drug screen |
|  | Brown et al., 2012 | 36.5 (8.2) | 34.3 (9.3) | 3.3 (1.5) | no | Yes- by clinical interview |
|  | Albrecht et al., 2013 | 37.9 (8.7) | 30.4 (7.3) | 4.28 (1.4) | no | Yes- by clinical interview and urine drug screen |
|  | Okita et al., 2016 | 38.6 (8.9) | 34.0 (9.1) | 3.9 (2.1) | yes | Yes- by clinical interview and urine drug screen |
|  | Wiers et al., 2017 | 31.5 (8.0) | 33.9 (9.1) | not mentioned | yes | Yes- by clinical interview and urine drug screen |
| D1 receptor | Dagher et al., 2001 | 30.6 (10.5) | 26 (7.4) | not mentioned | no | Yes- by clinical interview |
|  | Yasuno et al., 2007 | 28.5 (4.3) | 25.3 (4.3) | 5.9 (2.0) | no | Yes- by clinical interview |
